# P-1253. The ECOFF-Quest: TDM guided approach to piperacillin/tazobactam therapy in febrile neutropenia

**DOI:** 10.1093/ofid/ofaf695.1444

**Published:** 2026-01-11

**Authors:** Sara Ferin, Jacopo Angelini, Sarah Flammini, Giuseppe De Luise, Simone Giuliano, Carlo Tascini

**Affiliations:** University of Udine, Azienda Sanitaria Universitaria Friuli Centrale, Udine, Friuli-Venezia Giulia, Italy; Clinical Pharmacology and Toxicology Institute, University Hospital Friuli Centrale ASU FC, 33100 Udine, Italy, Udine, Friuli-Venezia Giulia, Italy; Infectious Diseases Division, Department of Medicine, University of Udine and Azienda Sanitaria Universitaria Friuli Centrale, Udine, Italy., Udine, Friuli-Venezia Giulia, Italy; University of Udine, Udine, Friuli-Venezia Giulia, Italy; Infectious Diseases Division, Department of Medicine, University of Udine and Azienda Sanitaria Universitaria Friuli Centrale, Udine, Italy, Udine, Friuli-Venezia Giulia, Italy; Udine University, UDINE, Friuli-Venezia Giulia, Italy

## Abstract

**Background:**

Piperacillin/tazobactam (PIP/TAZ) is a valuable empiric monotherapy for the initial treatment of febrile neutropenia, due to its broad-spectrum activity—including *Pseudomonas aeruginosa* (PA), *Escherichia coli* (EC), and *Klebsiella pneumoniae* (KP) - and favourable tolerability compared to other agents. However, the MERINO trial and emerging resistance threatens its use. This study evaluates pharmacokinetic/pharmacodynamic (PK/PD) data on continuous infusion (CI) of PIP/TAZ in febrile neutropenic patients, highlighting the role of Therapeutic Drug Monitoring (TDM) in optimizing its clinical use.

**Figure:**
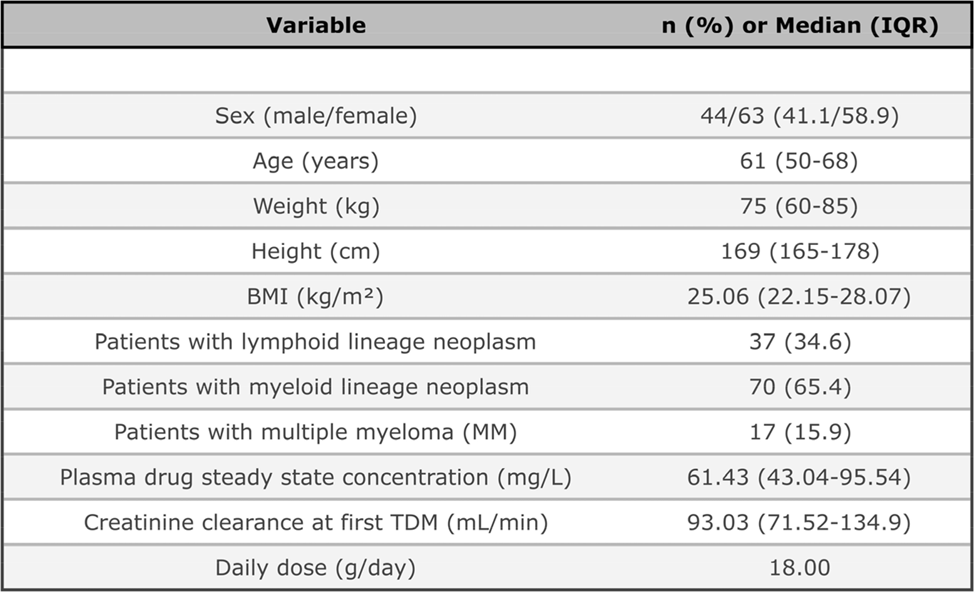
Demographic and clinical data at the first PIP/TAZ therapeutic drug monitoring.

**Figure:**
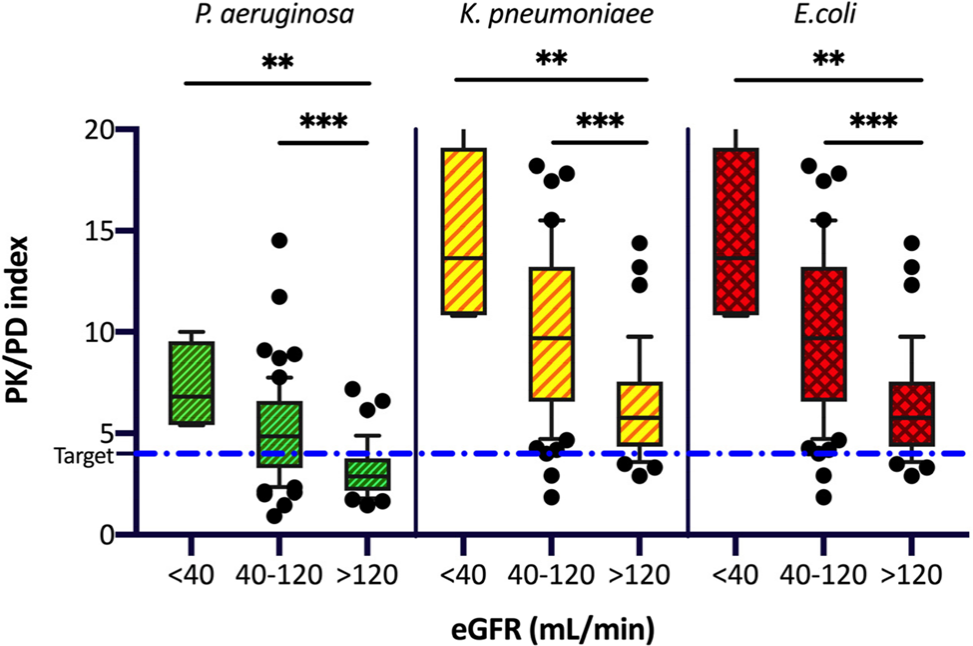
PK/PD index of piperacillin/tazobactam compared to the ECOFF for P. aeruginosa, K. pneumoniae, and E. coli across different classes of renal function

**Methods:**

This retrospective study included 107 patients treated with CI PIP/TAZ and undergoing TDM with at least one assay at steady state concentration (C_ss_), from January 2018 to February 2024. We investigated potential variables influencing drug clearance and the PK/PD target achievement (C_ss_ > 4xMIC for PIP) compared to the epidemiological cut-off value (ECOFF) for PA (16 mg/L), KP (8 mg/L) and EC (8 mg/L).

**Figure:**
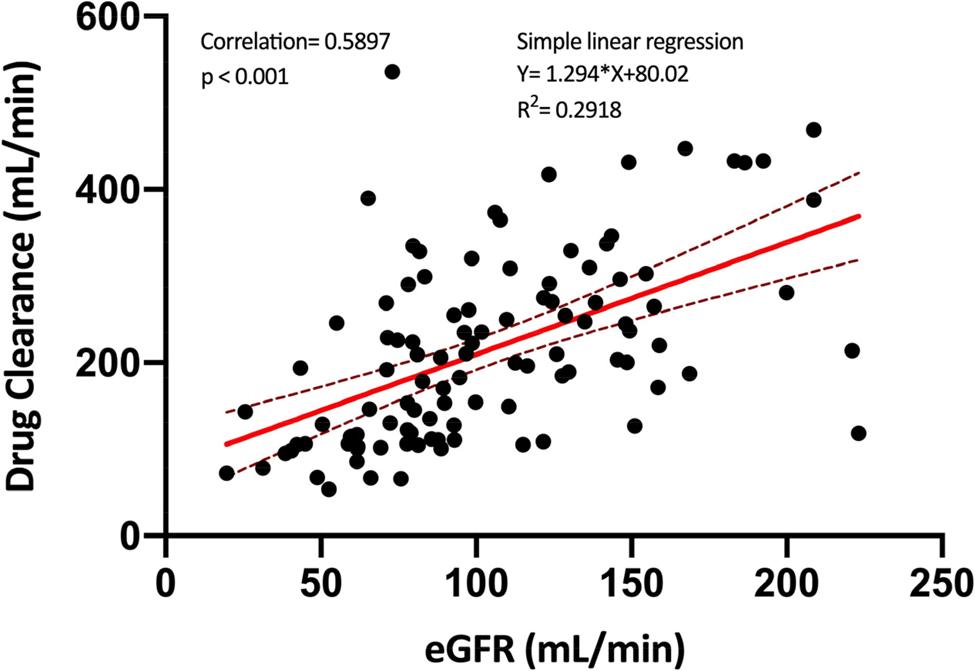
Correlation analysis between Creatinine clearance and drug clearance

**Figure:**
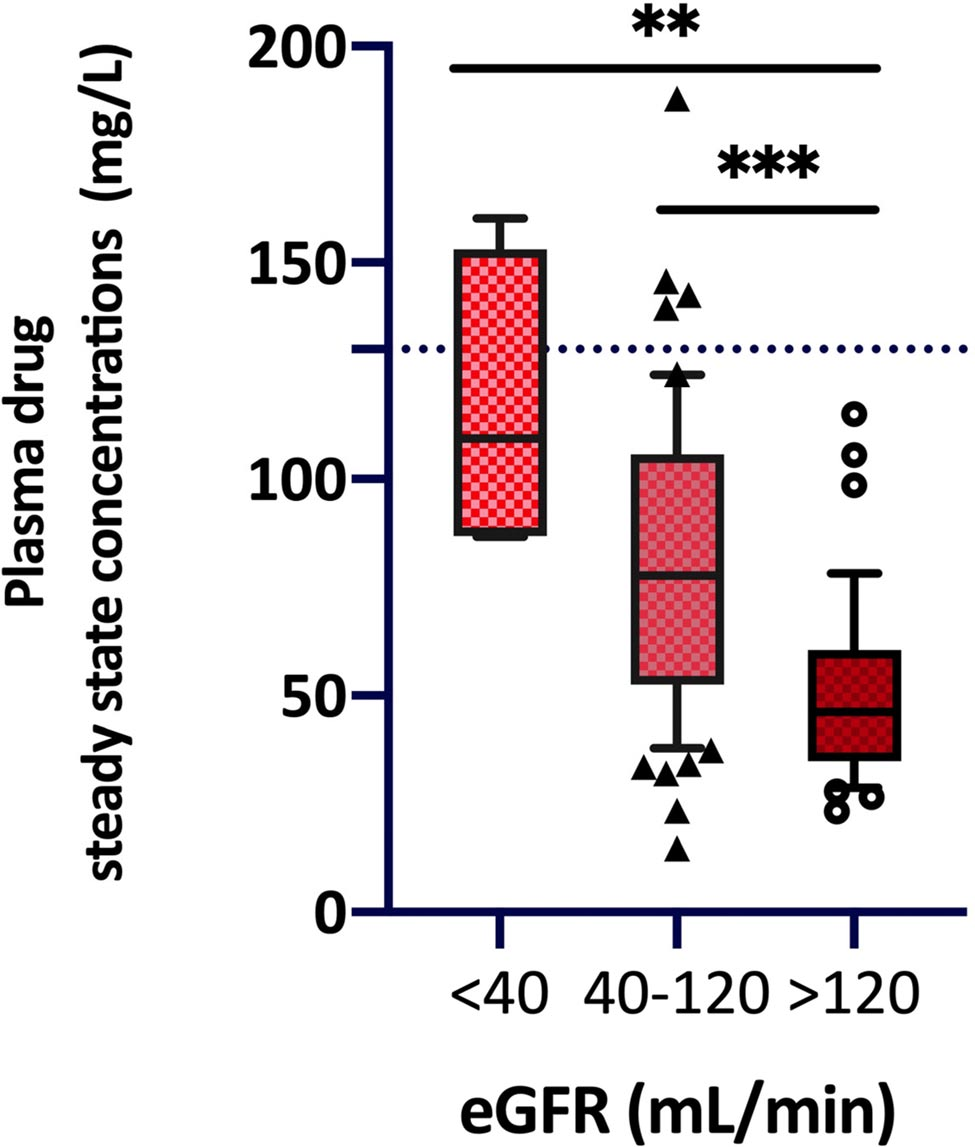
Steady-state drug concentrations stratified by renal function

**Results:**

Considering the ECOFF values, PK/PD target attainment resulted in 92.5% for EC and KP, and 46.73% for PA. 81.3% of patients with augmented renal clearance (eGFR >120mL/min) did not reach the PK/PD target for PA, accounting for the 65% of those who fell below the target. Dose-normalized PIP/TAZ exposure (C_ss_/Dose) was lower among hyperfiltrating patients (*p* value < 0.001). Glomerular filtration estimation was significantly associated but not fully predictive of drug clearance (*p* value < 0.001; R2=0.29), while BMI, sex, and nature of the hematologic disease were not correlated with plasma drug concentrations and its clearance.

**Conclusion:**

PIP/TAZ demonstrates a good empirical activity against EC and KP. However, PA remains the most critical pathogen, since even using CI administration, a minimum inhibitory concentration at the ECOFF threshold may result in potential underexposure to the drug, particularly in hyperfiltering patients. Notably, even in fragile neutropenic patients, circulating drug concentrations are permissive for dosage escalation, justifying the use of high-dose PIP/TAZ in such populations, including off-label dosing. However, drug clearance remains poorly predictable, highlighting the essential role of TDM to achieve the therapeutic target.

**Disclosures:**

Carlo Tascini, n/a, Angelini: Grant/Research Support|astellas: Grant/Research Support|astrazeneca: Grant/Research Support|biomerieux: Grant/Research Support|biotest: Grant/Research Support|gilead: Grant/Research Support|novartis: Grant/Research Support|thermofischer: Grant/Research Support|zambon: Grant/Research Support

